# Maternal plasma fatty acid patterns in mid-pregnancy and offspring epigenetic gestational age at birth

**DOI:** 10.1080/15592294.2022.2076051

**Published:** 2022-05-17

**Authors:** Giulietta S. Monasso, Trudy Voortman, Janine F. Felix

**Affiliations:** a The Generation R Study Group, Erasmus MC, University Medical Center Rotterdam, Rotterdam, the Netherlands; b Department of Pediatrics, Erasmus MC, University Medical Center Rotterdam, Rotterdam, the Netherlands; c Department of Epidemiology, Erasmus MC, University Medical Center, Rotterdam, The Netherlands

**Keywords:** Fatty acids, pregnancy, child, DNA methylation, epigenetic clock, epigenetic age, gestational age acceleration, cohort study

## Abstract

Maternal pregnancy fatty acid status is associated with child health. Epigenetic gestational age acceleration, referring to a discrepancy between chronological and epigenetic gestational age, may underlie these associations. Previous research suggests that analysing fatty acid patterns rather than individual fatty acids may overcome the caveat of missing synergistic or additive effects. Among 1226 mother-newborn pairs from the population-based Generation R Study, we examined the associations of three maternal plasma mid-pregnancy fatty acid patterns, identified by principal component analysis, with offspring epigenetic gestational age acceleration. This was estimated from cord blood DNA methylation data using the method developed by Bohlin. As a secondary analysis, we used the method developed by Knight to estimate epigenetic gestational age. The identified ‘high n-6 polyunsaturated fatty acid,’ ‘monounsaturated and saturated fatty acid’ and ‘high n-3 polyunsaturated fatty acid’ patterns were not associated with epigenetic gestational age acceleration in the main analyses. In sensitivity analyses restricted to 337 children born to mothers with more accurate pregnancy dating based on a regular menstrual cycle, a one standard-deviation-score higher maternal plasma ‘high n-3 polyunsaturated fatty acid’ pattern was associated with an epigenetic gestational age acceleration of 0.20 weeks (95% CI 0.06, 0.33), but only when using the Knight method. Thus, we found some evidence that a maternal plasma fatty acid pattern characterized by higher concentrations of n-3 polyunsaturated fatty acids may be associated with accelerated epigenetic gestational ageing. These findings depended on the method used and the accuracy of pregnancy dating and therefore need confirmation.

## Introduction

Fatty acids, especially essential fatty acids and their derivates, long-chain polyunsaturated fatty acids (LC-PUFAs), are transported from mother to foetus and are critical during foetal life [1,2]. They are involved in cell membrane synthesis and inflammatory processes and may modulate gene expression and protein function [1,3]. A number of observational studies have reported associations of maternal pregnancy fatty acid status and intake with various health outcomes in their children, including gestational age and preterm birth, asthma, body fat distribution and verbal intelligence [4–13].

A mechanism underlying the associations of fatty acid status during foetal life and health from birth onwards may be altered foetal DNA methylation [14–17]. Three previous pregnancy studies, using data from clinical trials, reported associations of LC-PUFA supplementation with offspring candidate-gene, global and regional methylation in neonatal blood [15,16,18]. Additionally, an epigenome-wide association study reported associations of maternal preconception, but not pregnancy, plasma fatty acid concentrations with offspring differential DNA methylation at 19 cytosine-phosphate-guanine sites (CpGs) in cord blood [14].

Fatty acid intake has also been associated with epigenetic ageing in four observational studies [19–22]. Epigenetic age can be established by ‘epigenetic clocks,’ biomarkers that predict age based on DNA methylation levels [23]. Positive age acceleration reflects an older epigenetic than chronological age. Negative age acceleration reflects a younger epigenetic than chronological age [23]. In adults, two large studies reported that omega-3 (n-3) PUFA intake was negatively correlated with age acceleration [19,20]. One small study reported that maternal intake of monounsaturated fatty acids (MUFAs) and saturated fatty acids (SFAs) during pregnancy was associated with offspring positive age acceleration, estimated from saliva sampled at birth [21]. A follow-up study embedded in the same observational study reported interactive associations of fatty acid classes with newborn age acceleration [22]. This suggests that synergistic or additive effects might be missed when analysing individual fatty acids. We and others previously showed that another approach to overcome this caveat may be the analysis of fatty acid patterns identified by principal component analysis [6,24]. This data-driven technique reduces the dimensionality of complex datasets [25]. It is not yet known whether maternal fatty acid patterns during pregnancy, established from plasma concentrations, are associated with offspring gestational age acceleration in cord blood.

In the current study, we aimed to examine the associations of three maternal pregnancy plasma fatty acid patterns with offspring epigenetic age acceleration, estimated from cord blood DNA methylation, among 1226 mother-newborn pairs. Previous studies have related both prenatal exposures considered to be beneficial and those considered to be detrimental to positive as well as negative age acceleration in cord blood. This indicates that the interpretation of a ‘beneficial’ effect may not be immediately apparent [26–31]. Therefore, we aimed to explore the direction of effect of associations as part of this study.

## Materials and methods

### Participants

This study was embedded in the Generation R Study, a population-based prospective cohort study from foetal life onwards in Rotterdam, the Netherlands [32]. The Medical Ethical Committee of Erasmus MC, University Medical Center Rotterdam, approved the study (MEC 198.782/2001/31). Pregnant women with an expected delivery date between April 2002 and January 2006 living in Rotterdam were eligible to participate. Written informed consent was obtained for all participants. We measured DNA methylation in cord blood in a European-ancestry subgroup of 1396 newborns. Child ethnicity was based on ethnicity of both parents according to the Statistics Netherlands. In the current study, we included 1226 of these newborns with information on maternal plasma mid-pregnancy fatty acid patterns available, after randomly excluding one sibling for each of the 12 (non-twin) sibling pairs in the data set. The analysis included only singleton children. Eleven newborns had missing data on CpGs required to calculate epigenetic age using our main method. These children were only included in analyses using a secondary method. Supplementary Figure 1 shows a flowchart of the study population.

### Maternal mid-pregnancy plasma fatty acid patterns and child gestational age acceleration

As previously described, non-fasting venous blood samples were collected in mid-pregnancy (median gestational age 20.5 (95% range 16.5–24.9) weeks) and subsequently transported and stored at −80 C in the regional laboratory, before being transported to the Division of Metabolic Diseases and Nutritional Medicine, Dr. von Hauner Children’s Hospital, Ludwig-Maximilians-University of Munich, Germany [33]. Gas chromatography was used to analyse the fatty acid composition of plasma phosphoglycerides. The average coefficient of variation was 15.7% [6]. We measured concentrations of 22 fatty acids, which were expressed in weight percentage (wt%) of total fatty acids in the chromatogram [6]. Previously, we applied principal component analysis on the wt% of these fatty acids, rather than on their concentrations, in order to reduce the dimensionality of our data. By using this data-driven mathematical approach, three principal components, or fatty acid patterns, were identified in maternal plasma, explaining the largest possible variation in the original variables (Supplementary Table 1) [6]. These were named after fatty acids with high (|≥0.20|) factor loadings for that particular plasma pattern, describing how strongly individual fatty acids contributed to a pattern [6]. The ‘high n-6 PUFA’ pattern was characterized by high factor loadings for n-6 PUFAs, the ‘MUFA and SFA’ pattern was characterized by high factor loadings for MUFAs and SFAs and the ‘high n-3 PUFA’ pattern was characterized by high factor loadings for n-3 PUFAs [6]. All mothers received an individual score on each of the plasma fatty acid patterns.

### DNA methylation data

We used the salting-out method to extract DNA from cord blood samples. Five-hundred nanograms of DNA were bisulphite converted using the EZ-96 DNA Methylation kit (Shallow) (Zymo Research Corporation, Irvine, USA). Samples were plated onto 96-well plates in no specific order. Samples were processed with the Illumina Infinium HumanMethylation450 BeadChip (Illumina Inc., San Diego, USA). Quality control and normalization were performed using the CPACOR workflow [34]. Probes with detection *p* values ≥1E-16 were set to be missing. Intensity values were quantile normalized. We removed arrays with technical problems, a call rate ≤95%, or a mismatch between the expected sex of participant and sex determined by chromosome X and Y probe intensities. Probes on the sex chromosomes were removed before the analyses. We used untransformed beta-values as measures of DNA methylation. The final dataset contained data on 458,563 CpGs.

### Gestational age estimation and gestational age acceleration

Pregnant mothers were seen for foetal ultrasound at our research centre in the first trimester of pregnancy [35]. During this visit, we established gestational age. If mothers had a known and reliable first day of the last menstrual period, and a regular menstrual cycle of 28 ± 4 d, the clinical estimate of gestational age was based on last menstrual period [35]. If mothers did not know the exact date of their last menstrual period, or had an irregular menstrual cycle, we established gestational age by ultrasound, which is the gold standard in clinical practice, but does not take into account variation in early foetal growth [35]. Consequently, measurement error may occur. Within the study population, we selected a subgroup of mothers with a known and reliable first day of the last menstrual period, and a regular menstrual cycle of 28 ± 4 d [35]. In this subgroup, a more accurate clinical estimate of gestational age could be made, based on last menstrual period (subgroup with ‘optimal’ pregnancy dating). These 337 mothers and their children were used for sensitivity analyses. We decided *a priori* to calculate epigenetic gestational age at birth (‘gestational age acceleration,’ in weeks) primarily based on the epigenetic clock of Bohlin using the GAprediction package 1.16.0 in R 3.6.1 [36,37]. This clock estimates gestational age from methylation levels at 96 CpGs from HumanMethylation450 BeadChip selected through Lasso-regression [36]. It is developed among newborns who are comparable to our full population, in terms of their characteristics [36,38]. We assessed both raw and residual gestational age acceleration. The raw measure, obtained by subtracting clinical from epigenetic gestational age, does not take into account the potential confounding effect of clinical age on epigenetic gestational age, which share variance [27]. We obtained the residual estimate from the residuals from a linear regression of epigenetic gestational age on clinical gestational age. By definition, residual acceleration is uncorrelated with clinical age [27]. As different clocks may capture different features of ageing, we used another commonly used cord-blood specific clock as secondary method (methylclock package 0.5.0 in R 3.6.1) [30,39,40]. This clock, developed by Knight, estimates gestational age from DNA methylation levels at 148 CpGs from HumanMethylation450 BeadChip, selected through elastic net regression [30]. As compared to the clock of Bohlin, this clock was developed among neonates who are less comparable to our study population [30,38]. More specifically, the Knight population had a mixed ethnic background and the average gestational age at birth was lower. Yet, as in our subgroup, pregnancy dating was based on last menstrual period.

### Covariates

We selected potential confounders based on literature. Maternal covariates included age (years), educational level (primary or secondary education versus university or higher), parity (nulliparous versus multiparous), pre-pregnancy body mass index (kg/m^2^), total daily energy intake during pregnancy (kcal, obtained from food-frequency questionnaires), folic acid supplementation during pregnancy (no use, started <10 weeks gestation, started periconceptionally), smoking during pregnancy (non-smoking or smoked until pregnancy was known, versus smoked throughout pregnancy), and alcohol consumption during pregnancy (no consumption or consumption until pregnancy was known, versus consumption throughout pregnancy). We adjusted for child sex and for gestational age at maternal blood sampling (weeks), as physiologically fatty acid concentrations decline during pregnancy [41]. We adjusted for batch effects by including plate number and used the ‘Salas’ reference set to estimate cell-type proportions in the ‘FlowSorted.CordBlood.Combined.450 K’ Bioconductor package. Briefly, this method estimates the relative proportions of six white blood cell subtypes (CD8+ T cells, CD4+ T cells, Natural Killer cells, B cells, Monocytes, Granulocytes) and nucleated Red Blood Cells, based on a standard reference of cord blood samples [42]. We considered sex- and gestational age-specific standard deviation scores (SDS) of birth weight as a potential mediator [43]. We obtained information on covariates from questionnaires sent out in pregnancy and from midwife and hospital records.

### Statistical analysis

First, we performed a non-response analysis, using Student’s t-tests, Mann–Whitney tests and Chi-square tests. Second, we compared characteristics of newborns included in the analyses, to those with DNA methylation data, but without data on maternal plasma fatty acid patterns or a sibling that was included. Third, we used linear regression to analyse the associations of continuous maternal plasma fatty acid pattern scores, which are statistically uncorrelated and standardized and can be interpreted as SDS, with raw and residual gestational age acceleration. The basic model was adjusted for batch, cell types, gestational age at blood sampling and sex. The main model was additionally adjusted for maternal age, education, body mass index, parity, energy intake, folic acid supplement use, smoking and alcohol consumption. This model was also run without cell-type adjustment (reduced main model), to examine the influence of variation in cell-type proportions [26]. In case of significant associations in the main model, we examined in the ‘mediator model’ whether our results changed after additional adjustment for birth weight SDS. We did not perform sex-stratified analyses, as the interaction terms of maternal plasma fatty acid pattern*sex were all not significant when added to the basic model. We examined potential non-linear associations between the maternal plasma fatty acid patterns and gestational age acceleration by adding a quadratic term for each plasma pattern to the main model. As this term was significant (p = 0.02) for the ‘high n-6 PUFA’ pattern in the residual gestational age acceleration model, we also assessed this plasma pattern in quintiles with the third quintile as reference. Fourth, we examined the associations of the three fatty acid patterns with clinical gestational age and epigenetic gestational age (main model). We used multiple imputation for missing covariates, using the Markov Chain Monte Carlo method. We created five datasets and reported pooled regression coefficients [44]. Statistical analyses were performed using the Statistical Package for the Social Sciences version 25.0 (SPSS IBM, Chicago, Illinois, United States). We adjusted for multiple testing using a Bonferroni correction and considered *p* values < (0.05/3 exposures), so <0.017, statistically significant.

## Results

### Subject characteristics

The study included 1226 mother-newborn pairs with data on maternal mid-pregnancy plasma fatty acid patterns and cord blood DNA methylation. Table 1 and Supplementary Table 2 show subject characteristics before and after imputation of covariates, respectively. Newborns had on average a slightly older clinical gestational age (median 40.3 weeks (95% range 36.7, 42.4)) than epigenetic gestational age (median 39.4 weeks (95% range 36.9, 40.8)). Consequently, the median raw gestational age acceleration was negative (Table 1). The correlation between clinical and epigenetic gestational age estimated by Bohlin’s clock was higher as compared to that estimated by Knight’s clock (Pearson’s correlation coefficients: 0.80 and 0.52, respectively) (Supplementary Figure 2). In the subgroup with optimal pregnancy dating, this correlation increased slightly for Knight’s clock (r = 0.59), but not for Bohlin’s clock (r = 0.81). The non-response analysis suggested that included newborns, on average, were not different from the 170 non-included newborns (Supplementary Table 3).

**Table 1.** Maternal and child characteristics based on non-imputed data (n = 1226)^1^.

**Maternal characteristics**	
Age, years	31.7 (4.2)
Educational level	
No or Primary	422 (35.0)
Higher	785 (65.0)
Parity	
Nulliparous	744 (60.7)
Multiparous	481 (39.3)
Pre-pregnancy body mass index, kg/m^2^	22.2 (18.4, 34.0)
Total daily energy intake (kcal)	2145 (495)
Gestational age at blood sampling, weeks	20.5 (18.6, 22.9)
Folic acid supplementation	
No	88 (8.8)
Started <10 weeks	312 (31.2)
Started periconceptionally	599 (60.0)
Smoking	
Non-smoker or smoked until pregnancy was known	960 (85.5)
Smoked throughout pregnancy	163 (14.5)
Alcohol consumption	
No consumption or consumption until pregnancy was known	500 (44.9)
Consumption throughout pregnancy	613 (55.1)
Pregnancy dating	
Based on last menstrual period	889 (72.5)
Based on ultrasound	337 (27.5)
‘high n-6 PUFA’ pattern^2^	0.005 (1.0)
‘MUFA and SFA’ pattern^2^	−0.006 (1.0)
‘high n-3 PUFA’ pattern^2^	−0.001 (1.0)
**Newborn characteristics**	
Gestational age at birth, weeks	40.3 (36.7, 42.4)
Epigenetic gestational age (Bohlin), weeks	39.4 (36.9, 40.8)
Raw gestational age acceleration (Bohlin), weeks	−0.89 (−2.70, 0.92)
Residual gestational age acceleration (Bohlin), weeks	0.03 (−1.24, 1.05)
Epigenetic gestational age (Knight), weeks	36.5 (32.4, 39.2)
Raw gestational age acceleration (Knight), weeks	−3.72 (−7.46, −1.13)
Residual gestational age acceleration (Knight), weeks	0.14 (−3.33, 2.52)
Sex	
Boy	623 (50.8)
Girl	603 (49.2)
Birth weight, grams	3580 (2523, 4505)

MUFA, monounsaturated fatty acid; PUFA, polyunsaturated fatty acid; SFA, saturated fatty acid.

^1^For the analyses based on Bohlin’s epigenetic clock, we excluded 11 newborns with missing values for some of the required CpGs, leaving 1215 children for analysis in the full population and 337 children in the subgroup with optimal pregnancy dating. Values are based on observed, not imputed data and are mean (SD) or median (95% range) for continuous variables and numbers (%) for categorical variables.

^2^Fatty acid pattern scores are standardized and can be interpreted as standard-deviation-scores.

### Maternal plasma fatty acid patterns during foetal development and gestational age acceleration

None of three maternal plasma fatty acid patterns, the ‘high n-6 PUFA’ pattern, the ‘MUFA and SFA’ pattern or the ‘high n-3 PUFA’ pattern, were associated with raw or residual gestational age acceleration in the main model, which was adjusted for batch, cell types, gestational age at blood sampling, sex, maternal age, education, body mass index, parity, energy intake, folic acid supplement use, smoking and alcohol consumption (all *P*-values ≥ 0.05; Table 2). This was not different in the subgroup with optimal pregnancy dating (Table 2). In secondary analyses based on Knight’s clock, none of the fatty acid plasma patterns were associated with gestational age acceleration either (Table 3). However, in the subgroup with optimal pregnancy dating, one SDS higher ‘high n-3 PUFA’ pattern score was associated with both raw (0.225 weeks, 95% confidence interval (CI): 0.07, 0.38) and residual (0.196 weeks, 95% CI: 0.06, 0.33) gestational age acceleration. These associations were similar after adjustment for birth weight SDS in the mediator model (Supplementary Table 4). Exclusion of the 11 newborns with missing CpGs for the Bohlin clock also did not change the results (Supplementary Table 5). The results from the reduced main model and basic model were largely similar to the main model (Supplementary Tables 6 and 7). No convincing non-linear association was observed when we analysed the ‘high n-6 PUFA’ pattern in quintiles (Supplementary Table 8). Associations of the three fatty acid patterns with clinical and epigenetic age (main models) can be found in Supplementary Table 9.

**Table 2.** Associations of maternal plasma fatty acid patterns in mid-pregnancy with offspring gestational age acceleration at birth by the epigenetic clock of Bohlin (main model).

	Raw acceleration^2^		Residual acceleration^3^	
	Difference (95% CI) in SDS	P value	Difference (95% CI) in SDS	P value
Full population (n = 1215)				
‘high n-6 PUFA’ pattern	0.000 (−0.06, 0.06)	1.00	−0.005 (−0.04, 0.03)	0.80
‘MUFA and SFA’ pattern	0.022 (−0.04, 0.08)	0.48	0.019 (−0.02, 0.06)	0.32
‘high n-3 PUFA’ pattern	0.038 (−0.02, 0.10)	0.23	0.009 (−0.03, 0.05)	0.63
Subgroup with optimal pregnancy dating (n = 336)				
‘high n-6 PUFA’ pattern	−0.019 (−0.13, 0.09)	0.74	−0.021 (−0.08, 0.04)	0.51
‘MUFA and SFA’ pattern	0.020 (−0.11, 0.15)	0.76	0.025 (−0.05, 0.10)	0.50
‘high n-3 PUFA’ pattern	0.083 (−0.04, 0.21)	0.19	0.046 (−0.03, 0.12)	0.21

CI, confidence interval; MUFA, monounsaturated fatty acid; PUFA, polyunsaturated fatty acid; SDS, standard deviation score; SFA, saturated fatty acid; SDS, standard deviation score.

Values represent regression coefficients (95% confidence interval) and reflect the difference in raw and residual gestational age acceleration at birth in weeks per increase of 1 standard deviation score in fatty acid pattern. Results are based on the main models, which were adjusted for child sex, batch effects (by including plate number), cell types and maternal age, education, pre-pregnancy body mass index, parity, total daily energy intake, gestational age at blood sampling and folic acid supplementation, smoking and alcohol consumption during pregnancy.

^1^For the analyses based on Bohlin’s epigenetic clock, we excluded 11 newborns with missing values for some of the required CpGs, leaving 1215 children for analysis in the full population and 336 of 337 children in the subgroup with optimal pregnancy dating.

^2^Raw gestational age acceleration was obtained by subtracting the clinical estimate of gestational age from epigenetic gestational age.

^3^Residual gestational age acceleration was calculated from the residuals from a regression model of epigenetic gestational age on clinical gestational age.

**Table 3.** Associations of maternal plasma fatty acid patterns in mid-pregnancy with offspring gestational age acceleration at birth by the epigenetic clock of Knight (main model).

	Raw acceleration^1^		Residual acceleration^2^	
	Difference (95% CI) in SDS	P value	Difference (95% CI) in SDS	P value
Full population (n = 1226)				
‘high n-6 PUFA’ pattern	−0.035 (−0.13, 0.06)	0.47	−0.040 (−0.12, 0.04)	0.34
‘MUFA and SFA’ pattern	−0.046 (−0.14, 0.05)	0.33	−0.049 (−0.13, 0.03)	0.25
‘high n-3 PUFA’pattern	0.027 (−0.07, 0.12)	0.57	0.005 (−0.08, 0.09)	0.91
Subgroup with optimal pregnancy dating (n = 337)				
‘high n-6 PUFA’ pattern	0.028 (−0.11, 0.17)	0.69	0.025 (−0.09, 0.14)	0.67
‘MUFA and SFA’ pattern	0.070 (−0.09, 0.23)	0.39	0.080 (−0.05, 0.22)	0.24
‘high n-3 PUFA’ pattern	0.225 (0.07, 0.38)	0.006*	0.196 (0.06, 0.33)	0.004*

CI, confidence interval; MUFA, monounsaturated fatty acid; PUFA, Polyunsaturated fatty acid; SDS, standard deviation score; SFA, saturated fatty acid; SDS, standard deviation score.

Values represent regression coefficients (95% confidence interval) and reflect the difference in raw and residual gestational age acceleration at birth in weeks per increase of 1 standard deviation score in fatty acid pattern. The main model was adjusted for adjusted for child sex, batch effects (by including plate number), cell types and maternal age, education, pre-pregnancy body mass index, parity, total daily energy intake, gestational age at blood sampling and folic acid supplementation, smoking and alcohol consumption during pregnancy. * Also significant after Bonferroni correction (0.05/3 exposures, thus 0.017).

^1^Raw gestational age acceleration was obtained by subtracting the clinical estimate of gestational age from epigenetic gestational age.

^2^Residual gestational age acceleration was calculated from the residuals from a regression model of epigenetic gestational age on clinical gestational age.

## Discussion

We found no evidence supporting associations of maternal plasma ‘high n-6 PUFA’ or ‘MUFA and SFA’ pattern scores with gestational age acceleration. Higher maternal plasma ‘high n-3 PUFA’ pattern scores may be associated with positive gestational age acceleration, indicating faster epigenetic than clinical gestational ageing. However, this association was only identified among newborns born to mothers with optimal pregnancy dating and when using the epigenetic clock of Knight, which was a secondary analysis.

Fatty acids are important for foetal growth and development [1]. Altered epigenetic gestational ageing may underlie the associations of maternal fatty acid status with child health [4–8]. We hypothesized that maternal plasma fatty acids are associated with gestational age acceleration. To overcome the caveat of missing synergistic or additive effects of fatty acids, we assessed potentially more informative fatty acid patterns, which were previously identified in the Generation R Study by principal component analysis. We aimed to explore the direction of effect of the associations as part of this study.

We observed that higher maternal plasma ‘high n-3 PUFA’ pattern scores were associated with positive gestational age acceleration by Knight’s clock in the subgroup with optimal pregnancy dating. Although no previous studies reported on plasma fatty acid patterns or concentrations and gestational age acceleration, two small Australian studies embedded in the same observational study assessed maternal dietary fatty acid intake in relation to offspring epigenetic gestational age acceleration in saliva, estimated by Horvath’s clock [21,22]. One of these, among 169 newborns, reported that maternal pregnancy dietary saturated and monounsaturated fat intake were associated with positive gestational age acceleration [21]. In a secondary analysis, palmitoleic acid (MUFA) was associated with positive gestational age acceleration, whereas total n-3 PUFA and α-linolenic acid (n–3 PUFA) were associated with negative gestational age acceleration [21]. In a follow-up study among 124 overlapping newborns, interactive associations between maternal dietary fatty acid classes in relation to offspring gestational age acceleration [22]. In two studies among non-pregnant adults, a negative correlation between n-3 intake and age acceleration was reported [19,20]. Overall, the direction of effect of our finding for the ‘high n-3 PUFA’ plasma pattern is in contrast to these previous studies. However, these studies are not directly comparable to our study. Firstly, fatty acid intake rather than patterns derived from plasma concentrations was assessed. Previously, a pregnancy study reported that dietary intake of eicosapentaenoic acid, docosahexaenoic acid, linoleic acid and arachidonic acid, which all contributed to the ‘high n-3 PUFA’ pattern, showed no to weak correlation (r = 0.05 to r = 0.24) with their plasma biomarker concentrations [45]. Secondly, the studies used other epigenetic clocks, which may capture different age-related biological processes [40]. Thirdly, the studies measured saliva rather than blood DNA methylation. Fourthly, two studies focused on adults, in whom the interpretation of the direction of effect of associations with epigenetic age acceleration may be different from that in newborns. Thus, confirmation of our finding in pregnancy studies with data on maternal plasma fatty acid plasma patterns is needed.

Prenatal exposures considered to be beneficial and those considered to be detrimental have both been related to both positive and negative gestational age acceleration, indicating that the interpretation of a ‘beneficial’ effect may not be immediately apparent [26–31]. Although some studies reported opposite or null associations, based on previous literature, higher maternal n-3 PUFAs status or intake overall seem to be associated with better offspring health [4–13]. A large Cochrane review reported that maternal n − 3 LC-PUFA supplement use during pregnancy was associated with a reduced risk of preterm birth and low birth weight [7]. Also, we previously reported associations of higher maternal plasma ‘high n-3 PUFA’ pattern scores with favourable body composition and serum lipid profiles in childhood [6]. Thus, our finding that higher maternal plasma ‘high n-3 PUFA’ pattern scores are associated with positive gestational age acceleration by Knight’s clock in the subgroup with optimal pregnancy dating could reflect better foetal growth or advanced maturation. Birth weight has been associated with positive gestational age acceleration, although not consistently [26,27,29]. Yet, birth weight did not explain our finding. Thus, the interpretation of gestational age acceleration in relation to higher maternal plasma ‘high n-3 PUFA’ pattern scores needs further study.

Overall, we observed no consistent associations of maternal plasma fatty acid patterns with gestational age acceleration. Accelerated epigenetic gestational ageing may not be a strong underlying mechanism for associations of maternal fatty acid status with childhood health. This does not necessarily rule out DNA methylation in itself being on the biological pathway between maternal fatty acid status and child health outcomes [46]. A previous epigenome-wide association study reported associations of maternal preconception plasma fatty acid concentrations with newborn DNA methylation at 19 CpGs [14]. Thus, DNA methylation changes, even though not reflective of altered ageing, may still underlie associations of fatty acid status during foetal life and childhood health. Alternatively, foetal DNA methylation changes may be epiphenomena rather than consequences of maternal fatty acid status [46]. Both alternatives could explain why we observed no associations in our main analysis. For the ‘MUFA and SFA’ pattern specifically, it may be that opposing effects of beneficial MUFAs and detrimental SFAs have balanced each other out, as suggested previously [6]. In a sensitivity analysis using the Knight method, we did find an association of the ‘high n-3 PUFA’ pattern with positive epigenetic gestational age acceleration. This finding needs replication in other studies. If confirmed, it would be interesting to examine if the epigenetic gestational age acceleration mediates associations with child health, such as metabolic outcomes. Future studies could also explore whether DNA methylation at other CpGs than those included in the examined clocks may mediate associations of maternal fatty acid levels and childhood health outcomes.

Our findings differed depending on which epigenetic clock we used. A previous study also reported different associations of various pregnancy exposures with gestational age acceleration by the clock of Bohlin versus that of Knight [28]. Similar to adult clocks, these neonatal clocks may capture different age-related biological processes [40]. Bohlin’s clock may capture aspects of age acceleration that are not a reflection of foetal adaptations to maternal fatty acid status, whereas Knight’s clock may capture adaptations in relation to maternal plasma ‘high n-3 PUFA’ pattern status. Similarly, our findings may also be related to the accuracy of pregnancy dating. The use of ultrasound may yield measurement errors and reduces biological variation in early foetal growth, potentially resulting from variation in maternal fatty acid status [47]. Therefore, clinical gestational age may have been predicted inaccurately for some children. Pregnancy dating based on last menstrual cycle may reduce measurement error and yield more precise clinical gestational age measurements. We indeed saw a slight increase in the correlation between clinical gestational age and epigenetic gestational age in the subgroup, especially when using epigenetic gestational age estimated by Knight’s clock. Consequently, we may have been able to better detect the positive finding for the ‘high n-3 PUFA’ pattern in this group.

A major strength of this study is its setting in an observational cohort. Further, we were the first study to report on fatty acid blood status, rather than intake, in relation to epigenetic age acceleration. Moreover, we examined fatty acid patterns, which take into account the intercorrelation of individual fatty acids. This study also has some limitations. First, as it was performed in an ethnically homogeneous European-ancestry subgroup, our findings may not be generalizable to other ancestries. Second, although the number of neonates with DNA methylation date was relatively large, we may have lacked power to find true associations of small magnitude. Third, our study population was relatively healthy, as indicated for example by the fact that almost all children were born at term. Limited variation in exposures or outcome may have prevented detection of true associations. Thus, replication in populations with more variation in maternal fatty acid status, and with different background characteristics, is needed. Fourth, the originally measured maternal plasma fatty acid concentrations do not necessarily reflect long-term exposure to fatty acids, which may be more relevant in relation to DNA methylation. We measured plasma fatty acids rather than measuring erythrocyte membrane fatty acid composition, which may better reflect the dietary intake of fats as it reflects a time frame of about 120 d rather than a few days [48]. Fifth, although we had data on many potentially confounding factors that may affect associations of maternal plasma fatty acid patterns with gestational age acceleration, residual confounding still could be an issue, as in any observational study. Sixth, although the associations of higher maternal pregnancy plasma ‘high n-3 PUFA’ pattern scores with positive gestational age acceleration in the subgroup remained after adjusting for multiple testing, this was a secondary analysis, which needs confirmation in future studies.

### Conclusions

Our findings suggest that maternal plasma ‘high n-6 PUFA’ and ‘MUFA and SFA’ patterns are not associated with gestational age acceleration. We found some evidence that plasma fatty acid patterns characterized by higher concentrations of n-3 polyunsaturated fatty acids may be associated with positive gestational age acceleration. These findings seem to depend on the method used to predict epigenetic gestational age and on the accuracy of pregnancy dating and therefore need confirmation in further studies.

## Supplementary Material

Supplemental Material

## Abbreviations

CI, confidence interval

MUFA, monounsaturated fatty acid

LC-PUFA, polyunsaturated fatty acid

PUFA, long-chain polyunsaturated fatty acid

SD, standard deviation

SFA, saturated fatty acid

SDS, standard deviation score

## Data sharing

Data described in the manuscript are available from the corresponding author on reasonable request, subject to the Generation R Study data access procedures.

## References

[1] Duttaroy AK. Transport of fatty acids across the human placenta: a review. Prog Lipid Res. 2009;48(1):52–61. 19041341 10.1016/j.plipres.2008.11.001

[2] Innis SM. Essential fatty acid transfer and fetal development. Placenta. 2005;26:S70–S5. 15837071 10.1016/j.placenta.2005.01.005

[3] Poudyal H, Panchal SK, Diwan V, et al. Omega-3 fatty acids and metabolic syndrome: effects and emerging mechanisms of action. Prog Lipid Res. 2011;50(4):372–387. 21762726 10.1016/j.plipres.2011.06.003

[4] Bisgaard H, Stokholm J, Chawes BL, et al. Fish oil–derived fatty acids in pregnancy and Wheeze and Asthma in Offspring. NEJM. 2016;375(26):2530–2539. 28029926 10.1056/NEJMoa1503734

[5] Hibbeln JR, Davis JM, Steer C, et al. Maternal seafood consumption in pregnancy and neurodevelopmental outcomes in childhood (ALSPAC study): an observational cohort study. Lancet. 2007;369(9561):578–585. 17307104 10.1016/S0140-6736(07)60277-3

[6] Voortman T, Tielemans MJ, Stroobant W, et al. Plasma fatty acid patterns during pregnancy and child’s growth, body composition, and cardiometabolic health: the Generation R Study. Clin Nutr. 2018;37(3):984–992. 28456538 10.1016/j.clnu.2017.04.006

[7] Middleton P, Gomersall JC, Gould JF, et al. Omega-3 fatty acid addition during pregnancy. Cochrane Database Syst Rev. 2018;11(11):CD003402. 30480773 10.1002/14651858.CD003402.pub3 PMC6516961

[8] Hoge A, Donneau AF, Dardenne N, et al. Impact of erythrocyte long-chain omega-3 polyunsaturated fatty acid levels in early pregnancy on birth outcomes: findings from a Belgian cohort study. J Perinatol. 2020;40(3):488–496. 31913325 10.1038/s41372-019-0573-9

[9] Nevins JEH, Donovan SM, Snetselaar L, et al. Omega-3 fatty acid dietary supplements consumed during pregnancy and lactation and child neurodevelopment: a systematic review. J Nutr. 2021;151(11):3483–3494. 34383914 10.1093/jn/nxab238 PMC8764572

[10] Voortman T, van den Hooven EH, Braun KV, et al. Effects of polyunsaturated fatty acid intake and status during pregnancy, lactation, and early childhood on cardiometabolic health: a systematic review. Prog Lipid Res. 2015;59:67–87. 26025302 10.1016/j.plipres.2015.05.001

[11] Makrides M, Best K, Yelland L, et al. A randomized trial of prenatal n-3 fatty acid supplementation and preterm delivery. N Engl J Med. 2019;381(11):1035–1045. 31509674 10.1056/NEJMoa1816832

[12] Ren X, Vilhjálmsdóttir BL, Rohde JF, et al. Systematic literature review and meta-analysis of the relationship between polyunsaturated and trans fatty acids during pregnancy and offspring weight development. Front Nutr. 2021;8(91). DOI:10.3389/fnut.2021.625596. PMC8027310 33842522

[13] Lee E, Kim H, Kim H, et al. Association of maternal omega-6 fatty acid intake with infant birth outcomes: Korean Mothers and Children’s Environmental Health (MOCEH). Nutr J. 2018;17(1):47. 29679982 10.1186/s12937-018-0353-y PMC5911376

[14] Robinson SL, Mumford SL, Guan W, et al. Maternal fatty acid concentrations and newborn DNA methylation. Am J Clin Nutr. 2020;111(3):613–621. 31858113 10.1093/ajcn/nqz311 PMC7049533

[15] Lee HS, Barraza-Villarreal A, Hernandez-Vargas H, et al. Modulation of DNA methylation states and infant immune system by dietary supplementation with ω-3 PUFA during pregnancy in an intervention study. Am J Clin Nutr. 2013;98(2):480–487. 23761484 10.3945/ajcn.112.052241 PMC3712555

[16] van Dijk SJ, Zhou J, Peters TJ, et al. Effect of prenatal DHA supplementation on the infant epigenome: results from a randomized controlled trial. Clin Epigenetics. 2016;8:114. 27822319 10.1186/s13148-016-0281-7 PMC5096291

[17] González-Becerra K, Ramos-Lopez O, Barrón-Cabrera E, et al. Fatty acids, epigenetic mechanisms and chronic diseases: a systematic review. Lipids Health Dis. 2019;18(1):178. 31615571 10.1186/s12944-019-1120-6 PMC6792183

[18] Lee HS, Barraza-Villarreal A, Biessy C, et al. Dietary supplementation with polyunsaturated fatty acid during pregnancy modulates DNA methylation at IGF2/H19 imprinted genes and growth of infants. Physiol Genomics. 2014;46(23):851–857. 25293351 10.1152/physiolgenomics.00061.2014 PMC4254937

[19] Lu AT, Quach A, Wilson JG, et al. DNA methylation GrimAge strongly predicts lifespan and healthspan. Aging (Albany NY). 2019;11(2):303–327. 30669119 10.18632/aging.101684 PMC6366976

[20] Quach A, Levine ME, Tanaka T, et al. Epigenetic clock analysis of diet, exercise, education, and lifestyle factors. Aging (Albany NY). 2017;9(2):419–446. 28198702 10.18632/aging.101168 PMC5361673

[21] Phang M, Ross J, Raythatha JH, et al. Epigenetic aging in newborns: role of maternal diet. Am J Clin Nutr. 2020;111(3):555–561. 31942922 10.1093/ajcn/nqz326

[22] Koemel NA, Senior AM, Dissanayake HU, et al. Maternal dietary fatty acid composition and newborn epigenetic aging—A Geometric Framework Approach. Am J Clin Nutr. 2022;115(1):118-127. 10.1093/ajcn/nqab318 34591100

[23] Horvath S. DNA methylation age of human tissues and cell types. Genome Biol. 2013;14:R115. 24138928 10.1186/gb-2013-14-10-r115 PMC4015143

[24] Anderson SG, Sanders TA, Cruickshank JK. Plasma fatty acid composition as a predictor of arterial stiffness and mortality. Hypertension. 2009;53(5):839–845. 19307467 10.1161/HYPERTENSIONAHA.108.123885

[25] Salem N, Hussein S. Data dimensional reduction and principal components analysis. Procedia Comput Sci. 2019;163:292–299.

[26] Khouja JN, Simpkin AJ, O’Keeffe LM, et al. Epigenetic gestational age acceleration: a prospective cohort study investigating associations with familial, sociodemographic and birth characteristics. Clin Epigenetics. 2018;10(86). DOI:10.1186/s13148-018-0520-1. PMC6020346 29983833

[27] Girchenko P, Lahti J, Czamara D, et al. Associations between maternal risk factors of adverse pregnancy and birth outcomes and the offspring epigenetic clock of gestational age at birth. Clin Epigenetics. 2017;8(9):49. 10.1186/s13148-017-0349-z PMC5422977 28503212

[28] Chen L, Wagner CL, Dong Y, et al. Effects of maternal vitamin D3 supplementation on offspring epigenetic clock of gestational age at birth: a post-hoc analysis of a randomized controlled trial. Epigenetics. 2020;15(8):830–840. 32089064 10.1080/15592294.2020.1734148 PMC7518698

[29] Bright HD, Howe LD, Khouja JN, et al. Epigenetic gestational age and trajectories of weight and height during childhood: a prospective cohort study. Clin Epigenetics. 2019;11(1):194. 31842976 10.1186/s13148-019-0761-7 PMC6916215

[30] Knight AK, Craig JM, Theda C, et al. An epigenetic clock for gestational age at birth based on blood methylation data. Genome Biol. 2016;17(1):206. 27717399 10.1186/s13059-016-1068-z PMC5054584

[31] Dieckmann L, Lahti-Pulkkinen M, Kvist T, et al. Characteristics of epigenetic aging across gestational and perinatal tissues. Clin epigenetics. 2021;13(1):97. 33926514 10.1186/s13148-021-01080-y PMC8082803

[32] Kooijman MN, Kruithof CJ, Van Duijn CM, et al. The Generation R Study: design and cohort update 2017. Eur J Epidemiol. 2016;31(12):1243–1264. 28070760 10.1007/s10654-016-0224-9 PMC5233749

[33] Kruithof CJ, Kooijman MN, van Duijn CM, et al. The Generation R Study: biobank update 2015. Eur J Epidemiol. 2014;29(12):911–927. 25527369 10.1007/s10654-014-9980-6

[34] Lehne B, Drong AW, Loh M, et al. A coherent approach for analysis of the Illumina HumanMethylation450 BeadChip improves data quality and performance in epigenome-wide association studies. Genome Biol. 2015;16:37. 25853392 10.1186/s13059-015-0600-x PMC4365767

[35] Gaillard R, Steegers EA, de Jongste JC, et al. Tracking of fetal growth characteristics during different trimesters and the risks of adverse birth outcomes. Int J Epidemiol. 2014;43(4):1140–1153. 24603318 10.1093/ije/dyu036 PMC4258770

[36] Bohlin J, Håberg SE, Magnus P, et al. Prediction of gestational age based on genome-wide differentially methylated regions. Genome Biol. 2016;17(1):207. 27717397 10.1186/s13059-016-1063-4 PMC5054559

[37] Bohlin J. GAprediction: prediction of gestational age with Illumina HumanMethylation450 data. R package version 1.16.0 ed2020.

[38] Simpkin AJ, Suderman M, Howe LD. Epigenetic clocks for gestational age: statistical and study design considerations. Clin Epigenetics. 2017;9(1):100. 28932320 10.1186/s13148-017-0402-y PMC5602851

[39] Pelegí-Sisó D, de Prado P, Ronkainen J, et al. methylclock: a bioconductor package to estimate DNA methylation age. Bioinformatics. 2021;37(12):1759–1760. 32960939 10.1093/bioinformatics/btaa825

[40] Liu Z, Leung D, Thrush K, et al. Underlying features of epigenetic aging clocks in vivo and in vitro. Aging Cell. 2020;19(10):e13229. 32930491 10.1111/acel.13229 PMC7576259

[41] Kawabata T, Kagawa Y, Kimura F, et al. Polyunsaturated fatty acid levels in maternal erythrocytes of Japanese women during pregnancy and after childbirth. Nutrients. 2017;9(3):245.

[42] Gervin K, Salas LA, Bakulski KM, et al. Systematic evaluation and validation of reference and library selection methods for deconvolution of cord blood DNA methylation data. Clin Epigenetics. 2019;11(1):125. 31455416 10.1186/s13148-019-0717-y PMC6712867

[43] Niklasson A, Ericson A, Fryer JG, et al. An update of the Swedish reference standards for weight, length and head circumference at birth for given gestational age (1977-1981). Acta Paediatr. 1991;80(8‐9):756–762. 10.1111/j.1651-2227.1991.tb11945.x 1957592

[44] Sterne JAC, White IR, Carlin JB, et al. Multiple imputation for missing data in epidemiological and clinical research: potential and pitfalls. BMJ. 2009;338:b2393. 19564179 10.1136/bmj.b2393 PMC2714692

[45] Madsen MTB, Bjerregaard AA, Furtado JD, et al. Comparisons of estimated intakes and plasma concentrations of selected fatty acids in pregnancy. Nutrients. 2019;11(3):568. 10.3390/nu11030568 PMC6470916 30845776

[46] Felix JF, Cecil C. Population DNA methylation studies in the developmental origins of health and disease (DOHaD) framework. J Dev Orig Health Dis. 2019;10(3):306–313. 30101736 10.1017/S2040174418000442

[47] Verburg BO, Steegers EA, De Ridder M, et al. New charts for ultrasound dating of pregnancy and assessment of fetal growth: longitudinal data from a population-based cohort study. Ultrasound Obstet Gynecol. 2008;31(4):388–396. 18348183 10.1002/uog.5225

[48] Fuhrman BJ, Barba M, Krogh V, et al. Erythrocyte membrane phospholipid composition as a biomarker of dietary fat. Ann Nutr Metab. 2006;50(2):95–102. 16373991 10.1159/000090496

