# Supplementary material for: Maternal plasma fatty acid patterns in mid-pregnancy and offspring epigenetic gestational age at birth
Source: Epigenetics. 2022 May 17;17(11):1562–72. doi: 10.1080/15592294.2022.2076051 (PMC9586633; doi:10.1080/15592294.2022.2076051)
Supplement: Supplemental Material [file KEPI_A_2076051_SM0705.docx]

**Maternal plasma fatty acid patterns in mid-pregnancy and offspring epigenetic gestational age at birth**

**Monasso et al.**

**Online Supplementary Material**

**Content**

Supplementary Figure 1. Flow chart of the study population

Supplementary Figure 2. Pearson’s correlation between clinical age and epigenetic age

Supplementary Table 1. Maternal plasma fatty acid patterns during mid-pregnancy

Supplementary Table 2. Maternal and child characteristics based on imputed data (n=1226)

Supplementary Table 3. Non-response analysis comparing newborns with DNA methylation available, with/without inclusion in the analyses

Supplementary Table 4. Associations of maternal plasma ‘high n-3 PUFA’ pattern in mid-pregnancy with offspring gestational age acceleration at birth by the epigenetic clock of Knight among 337 children born to mothers with optimal pregnancy dating (mediator model)

Supplementary Table 5. Associations of maternal plasma fatty acid patterns in mid-pregnancy with offspring gestational age acceleration at birth by the epigenetic clock of Knight (main model, after exclusion of newborns with missing CpGs for Bohlin clock)

Supplementary Table 6. Associations of maternal plasma fatty acid patterns in mid-pregnancy with offspring gestational age acceleration at birth by the epigenetic clock of Bohlin (reduced main model, n=1215)

Supplementary Table 7. Associations of maternal plasma fatty acid patterns in mid-pregnancy with offspring gestational age acceleration at birth by the epigenetic clock of Bohlin (basic model, n=1215

Supplementary Table 8. Associations of maternal plasma ‘high n-6 PUFA’ pattern in mid-pregnancy in quintiles with offspring residual gestational age acceleration at birth by the epigenetic clock of Bohlin (main model, n=1215)

Supplementary Table 9. Associations of maternal plasma fatty acid patterns in mid-pregnancy with offspring gestational age at birth (main model)

Live-born newborns and their mothers participating in the Generation R Study with offspring DNA methylation data

n = 1396

Exclusion: Mother-newborn pairs without data on maternal pregnancy fatty acid patterns in plasma

n = 158

Mother-newborn pairs with data on maternal pregnancy fatty acid patterns in plasma

n = 1238

Exclusion: Siblings

n = 12

Final cohort for analysis after imputation

All participants n = 1226 ^1,2^

Subgroup with optimal pregnancy dating^2,3^ n = 337

**Supplementary Figure 1. Flow chart of the study population**

1: The non-response analysis compared the characteristics of 1226 newborns included in the analyses, to those with DNA methylation data, but without data on maternal fatty acid patterns (n = 158), or a sibling that was included in the analyses (n=12).

2: For the analyses based on Bohlin’s epigenetic clock, we excluded 11 newborns with missing values for some of the required CpGs, leaving 1215 children for analysis in the full population and 336 children in the subgroup with optimal pregnancy dating

3: Based on a regular menstrual cycle of 28 ± 4 days and a known date of last menstrual period.


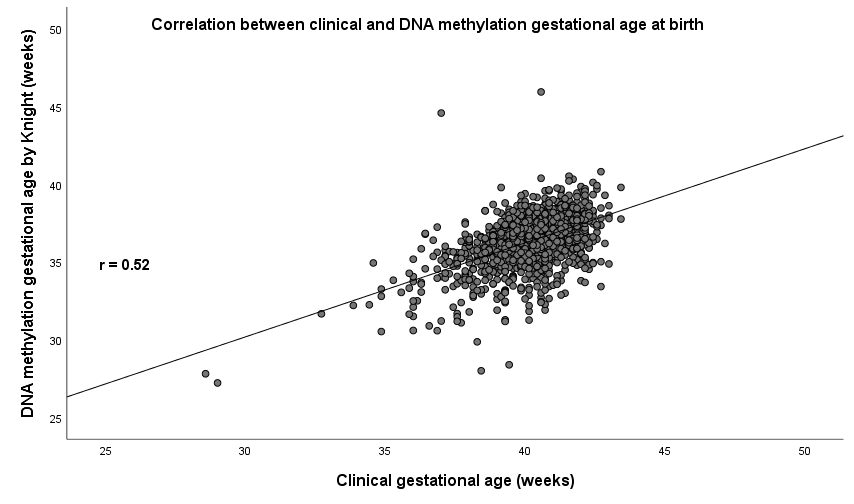

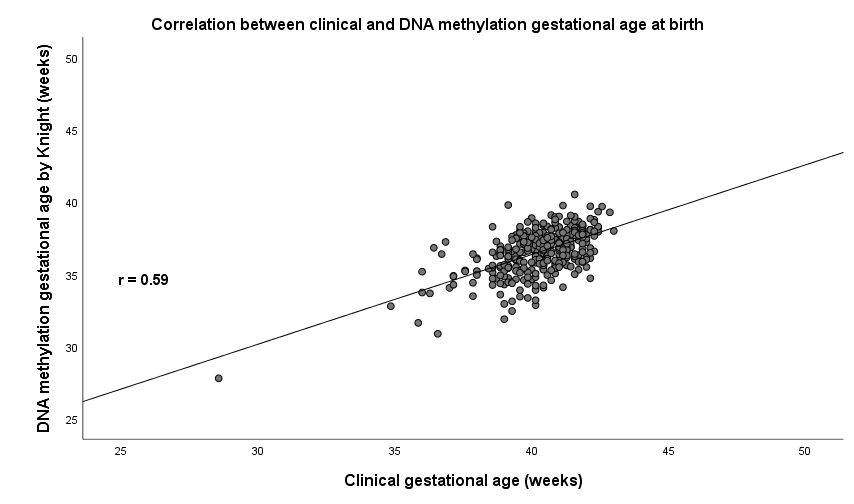

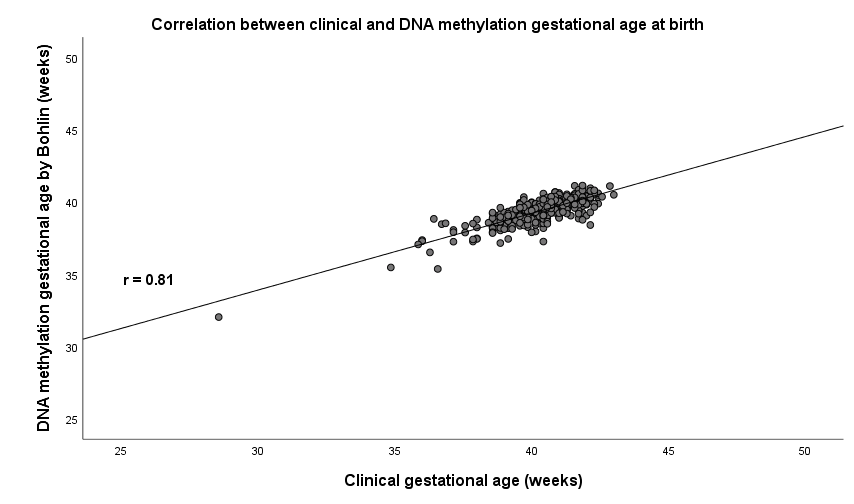

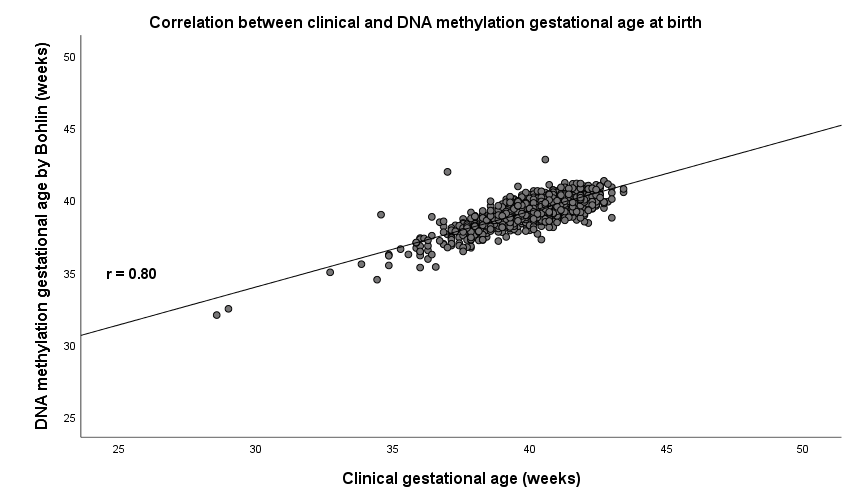


**D**

**C**

**A**

**B**

**A**

**Supplementary Figure 2. Pearson’s correlation between clinical age and epigenetic age**

Panels A-D show the correlation between clinical age (x-axes) and DNA methylation age (y-axes), estimated by the epigenetic gestational clocks of Bohlin or Knight.

**A**: The correlation between clinical and epigenetic gestational age using Bohlin’s clock calculated among all 1215 included children.

**B**: The correlation between clinical and epigenetic gestational age using Bohlin’s clock calculated among 336 children of mothers with optimal pregnancy dating based on having a regular menstrual cycle and a known first date of last menstrual period.

**C**: The correlation between clinical and epigenetic gestational age using Knight’s clock calculated among all 1226 included children.

**D**: The correlation between clinical and epigenetic gestational age using Knight’s clock calculated among 337 newborns of mothers with optimal pregnancy dating based on having a regular menstrual cycle and a known first date of last menstrual period.

Supplementary Table 1. Maternal plasma fatty acid patterns during mid-pregnancy

| **Fatty acids** | | | **Fatty acid patterns and factor loading** | | |
| --- | --- | --- | --- | --- | --- |
| **Type** | **Name** | **Lipid number** | **‘high n-6 PUFA’** | **‘MUFA and SFA’** | **‘high n-3 PUFA’** |
| Saturated |  |  |  |  |  |
|  | myristic acid | 14:0 | **0.23** | **0.30** | 0.06 |
|  | palmitic acid | 16:0 | 0.06 | **0.88** | 0.14 |
|  | margaric acid | 17:0 | -0.03 | **-0.31** | 0.03 |
|  | stearic acid | 18:0 | -0.08 | **-0.84** | -0.02 |
| Monounsaturated (cis) | pentadecanoic acid | 15:1n-5 | 0.01 | 0.16 | 0.14 |
|  | palmitoleic acid | 16:1n-7 | **0.43** | **0.63** | 0.16 |
|  | oleic acid | 18:1n-9 | 0.19 | **0.29** | **0.21** |
|  | vaccenic acid | 18:1n-7 | 0.00 | **0.28** | 0.08 |
|  | eicosenoic acid | 20:1n-9 | **-0.25** | **-0.31** | 0.09 |
| Polyunsaturated n-3 | α-linolenic acid | 18:3n-3 | -0.13 | -0.03 | 0.09 |
|  | eicosatrienoic acid | 20:3n-3 | 0.13 | -0.07 | 0.16 |
|  | eicosapentaenoic acid | 20:5n-3 | **-0.33** | 0.06 | **0.69** |
|  | docosapentaenoic acid | 22:5n-3 | 0.09 | -0.02 | **0.59** |
|  | docosahexaenoic acid | 22:6n-3 | **-0.39** | 0.06 | **0.67** |
| Polyunsaturated n-6 | linoleic acid | 18:2n-6 | **-0.45** | **-0.45** | **-0.67** |
|  | γ-linolenic acid | 18:3n-6 | **0.53** | 0.10 | 0.19 |
|  | eicosadienoic acid | 20:2n-6 | -0.06 | -0.04 | **-0.69** |
|  | dihomo-γ-linolenic acid | 20:3n-6 | **0.44** | **0.42** | -0.19 |
|  | arachidonic acid | 20:4n-6 | **0.40** | -0.15 | **0.30** |
|  | adrenic acid | 22:4n-6 | **0.85** | 0.03 | -0.08 |
|  | osbond acid | 22:5n-6 | **0.82** | 0.04 | -0.17 |
| Polyunsaturated n-9 | mead acid | 20:3n-9 | **0.53** | 0.12 | **0.37** |

MUFA, monounsaturated fatty acid; PUFA, polyunsaturated fatty acid; SFA, saturated fatty acid

We had data on concentrations of 22 fatty acids, which were expressed in weight percentage (wt%) of total fatty acids in the chromatogram. Subsequently, we applied principal component analysis on the wt% of these fatty acids. Three principal components (fatty acid patterns) were identified, explaining the largest possible variation in the original variables. These were named after fatty acids with high factor loadings for that particular pattern.

1: The factor loading describes how strongly individual fatty acids contributed to a pattern. **Factor loadings ≥|0.20| are considered hig**h.

**Supplementary Table 2. Maternal and child characteristics based on imputed data (n=1226)^1^**

| **Maternal characteristics** |  |
| --- | --- |
| Age, years | 31.7 (4.2) |
| Educational level |  |
| No or Primary | 429 (35.0) |
| Higher | 797 (65.0) |
| Parity |  |
| Nulliparous | 745 (60.8) |
| Multiparous | 481 (39.2) |
| Pre-pregnancy body mass index, kg/m^2^ | 22.3 (18.3, 33.4) |
| Total daily energy intake (kcal) | 2142 (496) |
| Gestational age at blood sampling, weeks | 20.5 (18.6, 22.9) |
| Folic acid supplementation |  |
| No | 113 (9.2) |
| Started <10 weeks | 379 (30.9) |
| Started periconceptional | 734 (59.9) |
| Smoking |  |
| Non-smoker or smoked until pregnancy was known | 1048 (85.5) |
| Smoked throughout pregnancy | 178 (14.5) |
| Alcohol consumption |  |
| No consumption or consumption until pregnancy was known | 550 (44.9) |
| Consumption throughout pregnancy | 676 (55.1) |
| Pregnancy dating |  |
| Based on last menstrual period | 889 (72.5) |
| Based on ultrasound | 337 (27.5) |
| ‘high n-6 PUFA’ pattern^2^ | 0.005 (1.0) |
| ‘MUFA and SFA’ pattern^2^ | -0.006 (1.0) |
| ‘high n-3 PUFA’ pattern^2^ | -0.001 (1.0) |
| **Newborn Characteristics** |  |
| Gestational age at birth, weeks | 40.3 (36.7, 42.4) |
| Epigenetic gestational age (Bohlin), weeks | 39.4 (36.9, 40.8) |
| Raw gestational age acceleration (Bohlin), weeks | -0.89 (-2.70, 0.92) |
| Residual gestational age acceleration (Bohlin), weeks | 0.03 (-1.24, 1.05) |
| Epigenetic gestational age (Knight), weeks | 36.5 (32.4, 39.2) |
| Raw gestational age acceleration (Knight), weeks | -3.72 (-7.46, -1.13) |
| Residual gestational age acceleration (Knight), weeks | 0.14 (-3.33, 2.52) |
| Sex |  |
| Boy | 623 (50.8) |
| Girl | 603 (49.2) |
| Birth weight, grams | 3580 (2523, 4505) |

MUFA, monounsaturated fatty acid; PUFA, polyunsaturated fatty acid; SFA, saturated fatty acid

1: For the analyses based on Bohlin’s epigenetic clock, we excluded 11 newborns with missing values for some of the required CpGs, leaving 1215 children for analysis in the full population and 337 children in the subgroup with optimal pregnancy dating. Values are based on observed, not imputed data and are mean (SD) or median (95% range) for continuous variables and numbers (%) for categorical variables.

2: Plasma fatty acid pattern scores are standardized and can be interpreted as standard-deviation-scores.

**Supplementary Table 3. Non-response analysis comparing newborns with DNA methylation available, with/without inclusion in the analyses^1,2^**

| **Maternal characteristics** | **Included (n=1226)** | **Not included (n=170)** | **P value^3^** |
| --- | --- | --- | --- |
| Age, years | 31.7 (4.2) | 31.6 (4.5) | 0.66 |
| Educational level |  |  | 0.64 |
| No or Primary | 422 (35.0) | 56 (32.9) |  |
| Higher | 785 (65.0) | 113 (67.1) |  |
| Parity |  |  | 0.81 |
| Nulliparous | 744 (60.7) | 101 (60.0%) |  |
| Multiparous | 481 (39.3) | 68 (40.0%) |  |
| Pre-pregnancy body mass index, kg/m^2^ | 22.2 (18.4, 34.0) | 22.7 (17.6, 33.4) | 0.31 |
| Total daily energy intake (kcal) | 2145 (495) | 2116 (523) | 0.51 |
| Folic acid supplementation |  |  | 0.76 |
| No | 88 (8.8) | 12 (7.1) |  |
| Started <10 weeks | 312 (31.2) | 40 (23.5) |  |
| Started periconceptional | 599 (60.0) | 89 (52.4) |  |
| Smoking |  |  | 0.70 |
| Non-smoker or smoked until pregnancy was known | 960 (85.5) | 136 (87.6) |  |
| Smoked throughout pregnancy | 163 (14.5) | 21 (12.4) |  |
| Alcohol consumption |  |  | 0.50 |
| No consumption or consumption until pregnancy was known | 500 (44.9) | 66 (46.5) |  |
| Consumption throughout pregnancy | 613 (55.1) | 91 (53.5) |  |
| Pregnancy dating |  |  | 0.49 |
| Based on last menstrual period | 889 (72.5) | 119 (70.0) |  |
| Based on ultrasound | 337 (27.5) | 51 (30.0) |  |
| **Newborn Characteristics** |  |  |  |
| Gestational age at birth, weeks | 40.3 (36.7, 42.4) | 40.3 (36.8, 42.1) | 0.12 |
| Epigenetic gestational age (Bohlin), weeks | 39.4 (36.9, 40.8) | 39.3 (36.5, 40.7) | 0.10 |
| Raw gestational age acceleration (Bohlin), weeks | -0.89 (-2.70, 0.92) | -0.80 (-2.80, 1.10) | 0.41 |
| Residual gestational age acceleration (Bohlin), weeks | 0.03 (-1.24, 1.05) | 0.02 (-1.81,1.24) | 0.93 |
| Epigenetic gestational age (Knight), weeks | 36.5 (32.4, 39.2) | 36.4 (32.4, 39.0) | 0.39 |
| Raw gestational age acceleration (Knight), weeks | -3.72 (-7.46, -1.13) | -3.64 (-6.99, -1.00) | 0.37 |
| Residual gestational age acceleration (Knight), weeks | 0.14 (-3.33, 2.52) | 0.18 (-3.42, 2.41) | 0.92 |
| Sex |  |  | 0.84 |
| Boy | 623 (50.8) | 85 (50.0) |  |
| Girl | 603 (49.2) | 85 (50.) |  |
| Birth weight, grams | 3580 (2523, 4505) | 3465 (2461, 4629) | 0.15 |

1: The non-response analysis compared the 1226 children who were included in the analyses to those who had information on DNA methylation data at birth available, but were not included because information on fatty acid patterns was missing (=158), or their sibling was included in the analyses (n=12).

2: Values are based on non-imputed data and are mean (SD) or median (95% range) for continuous variables and numbers (%) for categorical variables.

3: P-values for differences in subject characteristics between groups were calculated performing independent sample t-tests (normally distributed continuous variables), Mann Whitney tests (not normally distributed continuous variables) and chi-square tests (categorical variables).

Missing data: maternal education (n=19), parity (n=1), maternal body mass index (n=185), maternal total daily energy intake (n=109), maternal folic acid supplementation (n=227), maternal smoking (n=103), maternal alcohol consumption (n=113), birth weight (n=1).

|  | **Raw acceleration^2^** | | | **Residual acceleration^3^** | | |  |
| --- | --- | --- | --- | --- | --- | --- | --- |
|  | | **Difference (95% CI) in SDS** | **P value** | | **Difference (95% CI) in SDS** | **P value** | |
| ‘high n-3 PUFA’ pattern | | 0.238 (0.08, 0.40) | 0.004 | | 0.214 (0.08, 0.35) | 0.002 | |

Supplementary Table 4. Associations of maternal plasma ‘high n-3 PUFA’ pattern in mid-pregnancy with offspring gestational age acceleration at birth by the epigenetic clock of Knight among 337 children born to mothers with optimal pregnancy dating (mediator model)^1^

CI, confidence interval; SFA, saturated fatty acid; SDS, standard deviation score

Values represent regression coefficients (95% confidence interval) and reflect the difference in raw and residual gestational age acceleration at birth in weeks per increase of one standard deviation score in fatty acid pattern. The mediator model was adjusted for birth weight in addition to child sex, batch effects (by including plate number), cell types and maternal age, education, pre-pregnancy body mass index, parity, total daily energy intake, gestational age at blood sampling and folic acid supplementation, smoking and alcohol consumption during pregnancy.

1: Mothers with optimal pregnancy dating had a regular menstrual cycle of 28±4 days and in addition a known first day of their last menstrual cycle.

2: Raw gestational age acceleration was obtained by subtracting the clinical estimate of gestational age from epigenetic gestational age.

3: Residual gestational age acceleration was calculated from the residuals from a regression model of epigenetic gestational age on clinical gestational age.

Supplementary Table 5. Associations of maternal plasma fatty acid patterns in mid-pregnancy with offspring gestational age acceleration at birth by the epigenetic clock of Knight (main model, after exclusion of newborns with missing CpGs for Bohlin clock)

|  | **Raw acceleration^1^** | | **Residual acceleration^2^** | |
| --- | --- | --- | --- | --- |
|  | **Difference (95% CI) in SDS** | **P value** | **Difference (95% CI) in SDS** | **P value** |
| Full population (n=1226) |  |  |  |  |
| ‘high n-6 PUFA’ pattern | -0.031 (-0.13, 0.06) | 0.51 | -0.035 (-0.12, 0.05) | 0.41 |
| ‘MUFA and SFA’ pattern | -0.049 (-0.14, 0.04) | 0.30 | -0.052 (-0.13, 0.03) | 0.22 |
| ‘high n-3 PUFA’ pattern | 0.026 (-0.07, 0.12) | 0.58 | 0.003 (-0.08, 0.09) | 0.95 |
| Subgroup with optimal pregnancy dating (n=337) |  |  |  |  |
| ‘high n-6 PUFA’ pattern | 0.028 (-0.11, 0.17) | 0.70 | 0.025 (-0.09, 0.14) | 0.67 |
| ‘MUFA and SFA’ pattern | 0.078 (-0.08, 0.24) | 0.35 | 0.082 (-0.05, 0.22) | 0.24 |
| ‘high n-3 PUFA’ pattern | 0.226 (0.07, 0.39) | 0.005* | 0.196 (0.06, 0.33) | 0.004* |

CI, confidence interval; MUFA, monounsaturated fatty acid; PUFA, polyunsaturated fatty acid; SDS, standard deviation score; SFA, saturated fatty acid; SDS, standard deviation score

Values represent regression coefficients (95% confidence interval) and reflect the difference in raw and residual gestational age acceleration at birth in weeks per increase of one standard deviation score in fatty acid pattern. The main model was adjusted for adjusted for child sex, batch effects (by including plate number), cell types and maternal age, education, pre-pregnancy body mass index, parity, total daily energy intake, gestational age at blood sampling and folic acid supplementation, smoking and alcohol consumption during pregnancy. * Also significant after Bonferroni correction (0.05/3 exposures, thus 0.017).

1: Raw gestational age acceleration was obtained by subtracting the clinical estimate of gestational age from epigenetic gestational age.

2: Residual gestational age acceleration was calculated from the residuals from a regression model of epigenetic gestational age on clinical gestational age.

Supplementary Table 6. Associations of maternal plasma fatty acid patterns in mid-pregnancy with offspring gestational age acceleration at birth by the epigenetic clock of Bohlin (reduced main model, n=1215)^1^

|  | **Raw acceleration^2^** | | **Residual acceleration^3^** | |
| --- | --- | --- | --- | --- |
|  | **Difference (95% CI) in SDS** | **P value** | **Difference (95% CI) in SDS** | **P value** |
| ‘high n-6 PUFA’ pattern | -0.003 (-0.07, 0.06) | 0.93 | -0.021 (-0.06, 0.02) | 0.29 |
| ‘MUFA and SFA’ pattern | 0.026 (-0.04, 0.09) | 0.41 | 0.015 (-0.02, 0.05) | 0.45 |
| ‘high n-3 PUFA’ pattern | 0.041 (-0.02, 0.10) | 0.20 | 0.014 (-0.03, 0.05) | 0.47 |

CI, confidence interval; MUFA, monounsaturated fatty acid; PUFA, polyunsaturated fatty acid; SDS, standard deviation score; SFA, saturated fatty acid

Values represent regression coefficients (95% confidence interval) and reflect the difference in raw and residual gestational age acceleration at birth in weeks per increase of one standard deviation score in fatty acid pattern. The reduced main model was adjusted for child sex, batch effects (by including plate number) and maternal age, education, pre-pregnancy body mass index, parity, total daily energy intake, gestational age at blood sampling and folic acid supplementation, smoking and alcohol consumption during pregnancy.

1: For the analyses based on Bohlin’s epigenetic clock, we excluded 11 newborns with missing values for some of the required CpGs.

2: Raw gestational age acceleration was obtained by subtracting the clinical estimate of gestational age from epigenetic gestational age.

3: Residual gestational age acceleration was calculated from the residuals from a regression model of epigenetic gestational age on clinical gestational age.

Supplementary Table 7. Associations of maternal plasma fatty acid patterns in mid-pregnancy with offspring gestational age acceleration at birth by the epigenetic clock of Bohlin (basic model, n=1215)^1^

|  | **Raw acceleration^2^** | | | | **Residual acceleration^3^** | | | |
| --- | --- | --- | --- | --- | --- | --- | --- | --- |
|  | **Difference (95% CI) in SDS** | | **P value** | | **Difference (95% CI) in SDS** | | **P value** | |
| ‘high n-6 PUFA’ pattern | 0.053 (0.00, 0.11) | 0.05 | | 0.022 (-0.01, 0.05) | | 0.17 | |  |
| ‘MUFA and SFA’ pattern | 0.039 (-0.02, 0.10) | 0.17 | | 0.022 (-0.01, 0.06) | | 0.19 | |  |
| ‘high n-3 PUFA’ pattern | 0.020 (-0.04, 0.08) | 0.49 | | -0.003 (-0.04, 0.03) | | 0.88 | |  |

CI, confidence interval; MUFA, monounsaturated fatty acid; PUFA, polyunsaturated fatty acid; SDS, standard deviation score; SFA, saturated fatty acid

Values represent regression coefficients (95% confidence interval) and reflect the difference in raw and residual gestational age acceleration at birth in weeks per increase of one standard deviation score in fatty acid pattern. The basic model was adjusted for child sex, batch effects (by including plate number) cell types and gestational age at blood sampling.

We considered p-values after Bonferroni correction for 3 exposures (0.05/3 exposures, thus 0.017) significant.

1: For the analyses based on Bohlin’s epigenetic clock, we excluded 11 newborns with missing values for some of the required CpGs.

2: Raw gestational age acceleration was obtained by subtracting the clinical estimate of gestational age from epigenetic gestational age.

3: Residual gestational age acceleration was calculated from the residuals from a regression model of epigenetic gestational age on clinical gestational age.

Supplementary Table 8. Associations of maternal plasma ‘high n-6 PUFA’ pattern in mid-pregnancy in quintiles with offspring residual gestational age acceleration at birth by the epigenetic clock of Bohlin (main model, n=1215)^1^

|  | **Residual acceleration^2^** | |
| --- | --- | --- |
|  | **Difference (95% CI) in SDS** | **P value** |
| ‘high n-6 PUFA’ pattern |  |  |
| Q1 | -0.045 (-0.15, 0.06) | 0.40 |
| Q1 | -0.017 (-0.12, 0.09) | 0.75 |
| Q3 | *Reference* |  |
| Q4 | -0.065 (-0.17, 0.04) | 0.23 |
| Q5 | -0.065 (-0.17, 0.04) | 0.24 |

CI, confidence interval; PUFA, polyunsaturated fatty acid; SDS, standard deviation score

Values represent regression coefficients (95% confidence interval) and reflect the difference in residual gestational age acceleration at birth in weeks per increase of one standard deviation score in fatty acid pattern. The main model was adjusted for child sex, batch effects (by including plate number), cell types and maternal age, education, pre-pregnancy body mass index, parity, total daily energy intake, gestational age at blood sampling and folic acid supplementation, smoking and alcohol consumption during pregnancy.

1: For the analyses based on Bohlin’s epigenetic clock, we excluded 11 newborns with missing values for some of the required CpGs.

2: Residual gestational age acceleration was calculated from the residuals from a regression model of epigenetic gestational age on clinical gestational age.

**Supplementary Table 9. Associations of maternal plasma fatty acid patterns in mid-pregnancy with offspring gestational age at birth (main model)^1^**

|  | **Clinical gestational age** | | **Bohlin gestational age** | | | **Knight gestational age** | | |  |
| --- | --- | --- | --- | --- | --- | --- | --- | --- | --- |
|  | **Difference (95% CI) in SDS** | **P value** | | **Difference (95% CI) in SDS** | **P value** | | **Difference (95% CI) in SDS** | **P value** | |
| Full population (n=1215) |  |  | |  |  | |  |  | |
| ‘high n-6 PUFA’ pattern | -0.013 (-0.11, 0.08) | 0.79 | | -0.010 (-0.07, 0.05) | 0.75 | | -0.048 (-0.15, 0.05) | 0.34 | |
| ‘MUFA and SFA’ pattern | -0.007 (-0.10, 0.09) | 0.88 | | 0.015 (-0.05, 0.08) | 0.62 | | -0.053 (-0.15, 0.04) | 0.28 | |
| ‘high n-3 PUFA’ pattern | -0.057 (-0.14, 0.04) | 0.25 | | -0.022 (-0.09, 0.04) | 0.48 | | -0.029 (-0.13, 0.07) | 0.56 | |
| Subgroup with optimal pregnancy dating (n=336) |  |  | |  |  | |  |  | |
| ‘high n-6 PUFA’ pattern | -0.008 (-0.19, 0.17) | 0.93 | | -0.025 (-0.13, 0.09) | 0.66 | | 0.021 (-0.13, 0.18) | 0.79 | |
| ‘MUFA and SFA’ pattern | 0.025 (-0.18, 0.24) | 0.81 | | 0.030 (-0.10, 0.16) | 0.64 | | 0.096 (-0.08, 0.27) | 0.29 | |
| ‘high n-3 PUFA’ pattern | -0.073 (-0.28, 0.13) | 0.49 | | 0.007 (-0.12, 0.13) | 0.91 | | 0.152 (-0.02, 0.33) | 0.09 | |

CI, confidence interval; MUFA, monounsaturated fatty acid; PUFA, Polyunsaturated fatty acid; SDS, standard deviation score; SFA, saturated fatty acid; SDS, standard deviation score

1: For the analyses based on Bohlin’s epigenetic clock, we excluded 11 newborns with missing values for some of the required CpGs.
